# Comparing low-dose (DART) and enhanced low-dose dexamethasone regimens in preterm infants with bronchopulmonary dysplasia

**DOI:** 10.3389/fped.2023.1261316

**Published:** 2023-10-31

**Authors:** Heba Mohamed Al-taweel, Ismail Sabry Ismail Abdelhady, Nasreen Irfan, Fadi Al Khzzam, Abdullah Kamal, Sudheer Babu Kurunthattil Thazhe, Mohammad A. A. Bayoumi, Ashraf Gad

**Affiliations:** ^1^ Pharmacy Department, Women’s Wellness and Research Center, Hamad Medical Corporation, Doha, Qatar; ^2^ Neonatal Intensive Care Unit, Women’s Wellness and Research Center, Hamad Medical Corporation, Doha, Qatar; ^3^ Pediatric Department, Children’s Hospital of Eastern Ontario and Ottawa Hospital, University of Ottawa, Ottawa, ON, Canada; ^4^ Pediatric Department, Weill Cornell Medicine-Qatar, Doha, Qatar

**Keywords:** postnatal steroids, dexamethasone, extremely low birth weight, bronchopulmonary dysplasia, chronic lung disease of prematurity, Respiratory distress syndrome, preterm (birth), neonatal intensive care unit (NICU)

## Abstract

**Introduction:**

Determining the optimal dexamethasone dosage for facilitating extubation in extremely low birth weight (ELBW) infants with bronchopulmonary dysplasia (BPD) remains uncertain. This study aims to compare the effectiveness of low-dose (DART) and enhanced low-dose dexamethasone regimens in achieving successful extubation in these infants.

**Methods:**

We conducted a retrospective cohort study at the Women's Wellness and Research Center (WWRC) involving ELBW infants who received dexamethasone for BPD prevention or treatment, or for extubation between January 1st, 2015, and December 31st, 2019. Our goal was to assess successful extubation within various time points of treatement.

**Results:**

A total of 77 patients, matched in gestational age and BW, were enrolled in the study, receiving a total of 121 dexamethasone courses. Low-dose dexamethasone courses were administered 75 times to 49 infants, while 46 courses of enhanced low-dose were given to 28 infants. Treatment commenced at 30.8 ± 3.4 weeks post-menstrual age, compared to 32.1 ± 2.5 weeks in the enhanced low-dose group (*p* = 0.014). The median (IQR) course duration was seven (3–10) days in the low-dose group, while it was 10 (8–14) days in the enhanced low-dose group (*p* < 0.001). The median (IQR) course dose was 0.73 (0.53–0.86) mg/kg in the low-dose group and 1.27 (0.97–2.05) mg/kg in the enhanced low-dose group (*p* < 0.001). There were no differences in extubation success at any time point between the two groups at 72 h and seven days after treatment initiation, by course completion, and within seven days after treatment completion. However, regression analysis identified several predictors of successful extubation; baseline FiO_2_, course duration, and duration of invasive mechanical ventilation were negatively associated with successful extubation at various time points, while received dose per kg and cumulative dose positively correlated with successful extubation at different time points. No significant differences were observed in secondary outcomes, including death or BPD.

**Conclusion:**

The choice between low-dose and enhanced low-dose dexamethasone regimens may not significantly impact extubation success. However, careful consideration of dosing, ventilation status, and treatment duration remains crucial in achieving successful extubation. This study highlights the need for personalized dexamethasone therapy in ELBW infants.

## Introduction

Bronchopulmonary dysplasia (BPD) is a common neonatal respiratory disease affecting preterm neonates, particularly those who are extremely low birth weight (ELBW) (birth weight less than 1,000 g) (1–4). BPD is a significant cause of morbidity and mortality in this population, with prominent outcomes including cardiovascular and neurodevelopmental delay (5–7). Several factors contribute to BPD in these infants, including perinatal inflammation, sepsis, necrotizing enterocolitis (NEC), and the need for mechanical ventilation and oxygen supplementation (6, 7). These conditions collectively promote damage and inflammation in the alveoli and airways, resulting in abnormal lung development and BPD onset (4). Despite neonatal care advancements, BPD prevalence remains high, and previous therapies primarily focused on mitigating its effects rather than prevention or cure.

In recent years, studies have explored therapies aimed at reducing the BPD prevalence in preterm infants and improving their short-term and long-term outcomes (3–5). Among these therapies, postnatal steroids (PNS) have gained significant attention for their ability to reduce lung inflammation (8–14). PNS achieve this by suppressing lymphocytes and enhancing the effects of anti-inflammatory cytokines (9). PNS therapy is commonly used to treat and prevent BPD and to facilitate extubation from mechanical ventilation (12, 13). However, their use in neonates, especially preterm infants, is associated with potential adverse effects, including impaired growth and development, an increased risk of infection, and neurodevelopmental problems (2, 14–17, 18).

Studies have assessed the efficacy and safety of PNS, particularly dexamethasone, with various doses or durations in neonates (19–22). The most commonly studied protocol is the DART (Dexamethasone: A Randomized Trial) protocol, which involves a lower dose (total 0.89 mg/kg) over 10 days for the treatment of BPD in preterm infants (23). Despite its long-standing use, BPD remains a significant cause of neonatal morbidity. In recent years, clinicians have started to explore different dosing regimens and durations for dexamethasone to evaluate potential benefits (20, 21, 24).

The efficacy and safety of low-dose dexamethasone (0.08 mg/kg/day for seven days) compared to high dose (0.5 mg/kg/day for three days followed by 0.3 mg/kg/day for four days) was evaluated in a study involving preterm neonates with BPD (25). The study found that low-dose dexamethasone therapy was associated with lower incidence of adverse effects, including growth failure and hypertension, compared to conventional dexamethasone therapy with similar pulmonary outcomes. Another study used early dexamethasone therapy at a higher overall dose (0.5 mg/kg/day for seven days) vs. placebo (0.9% normal saline) (21). This study found that dexamethasone improved short-term pulmonary outcome compared to placebo, with no reported serious adverse effects in either group.

These findings underscore the variability in current research regarding the optimal duration and dose for dexamethasone. The American Academy of Pediatrics (AAP) state that doses ≥0.5 mg/kg are associated with adverse outcomes. However, insufficient data is available on the potential benefits and risks of lower doses of dexamethasone in neonates (26).

Overall, studies suggest that the optimal dexamethasone regimen is unclear. However, doses higher than those recommended in the low-dose DART protocol show promise in effectively reducing BPD without significant differences in adverse events. Neonatologists often favor the DART regimen due to the lack of established evidence for alternative dexamethasone dose regimens.

The objective of this study is to conduct a retrospective analysis to investigate the clinical efficacy of low-dose dexamethasone dose (DART protocol) compared to enhanced low-dose dexamethasone regimens in facilitating extubation, and to examine their effects on significant neonatal outcomes in ELBW infants with evolving or established BPD.

## Materials and methods

### Study population

This retrospective cohort study was conducted at the Women's Wellness and Research Center (WWRC) (former Women's Hospital) in Doha, Qatar. The study included ELBW infants who received dexamethasone for the prevention or treatment of BPD. The study period lasted from January 2015 to December 2019.

We identified ELBW infants who were intubated for more than two weeks thus qualifying for PNS dexamethasone treatment per unit protocol. Pharmacy records were reviewed to identify those infants who were administered the dexamethasone regimen, as prescribed by the attending physician's discretion. We categorized infants into two groups based on the dexamethasone dosage they received. The first group received a low dose as per the DART protocol, which amounted to a cumulative dose of 0.89 mg/kg spread over 10 days. The second group received a cumulative dose exceeding 0.89 mg/kg, which is referred to as the “enhanced low-dose dexamethasone”. To determine the doses of dexamethasone received, we considered the actual doses administered, regardless of the ordered dose. Dosing weight was based on the weight recorded at the time of prescription.

**Figure 1:**
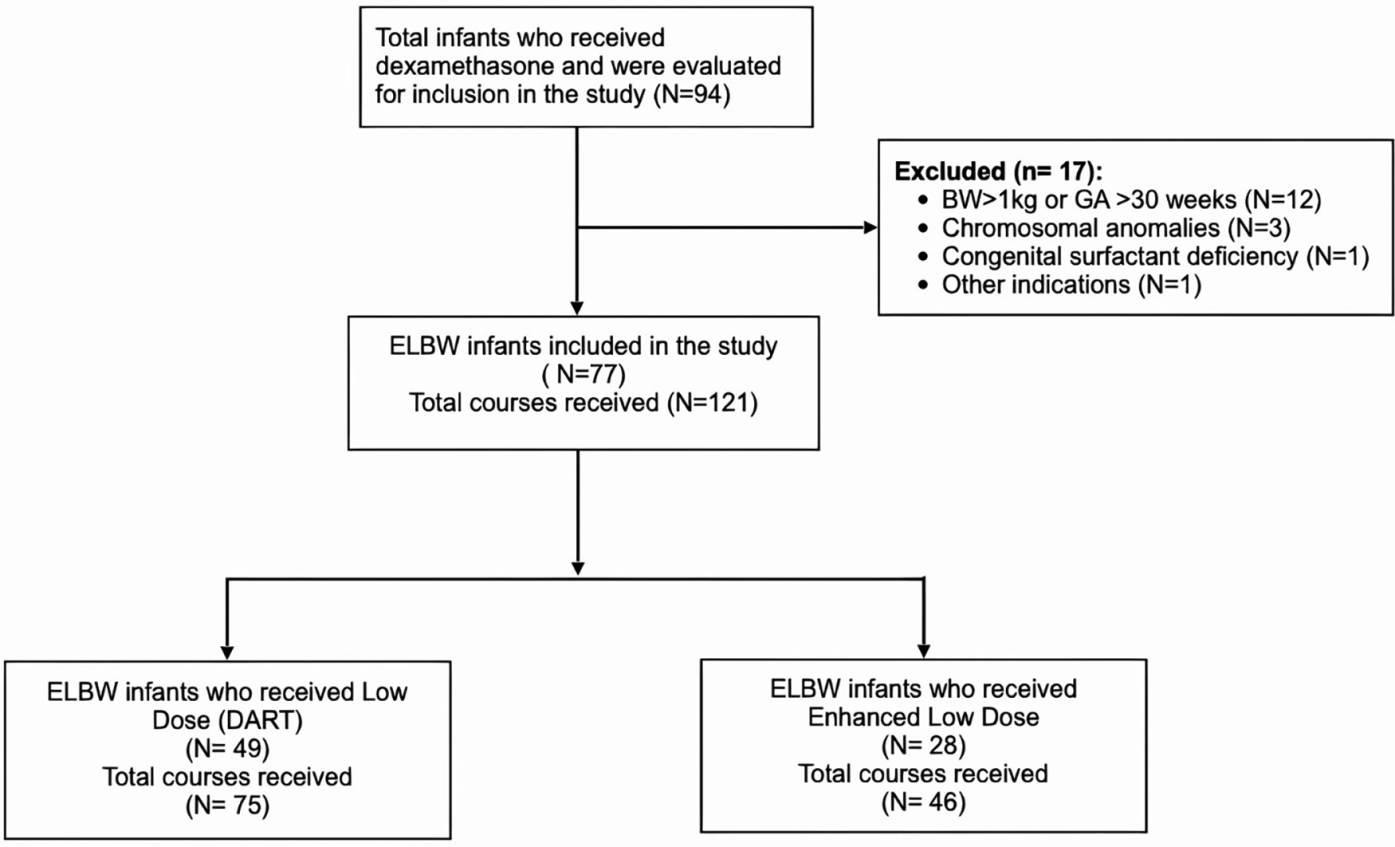
Flow diagram of inclusion criteria and dexamethasone dose regimens. BW, birth weight; GA, gestational age; ELBW, extremely low birth weight. DART, dexamethasone; a randomized trial.

We identified a total of 94 infants as receiving dexamethasone; however, 17 infants were excluded from the study. The exclusion criteria were as follows: 12 infants had higher gestational age and birth weight (BW), three infants had chromosomal anomalies, one infant received steroids during subglottic correction surgery, and one infant had congenital surfactant deficiency (Figure 1). Infants who received hydrocortisone were also excluded from the study.

This study was approved and received ethical approval by the WWRC Medical Research Center (MRC)/Institutional Review Board (IRB) under protocol number MRC-01-19-301. The study did not receive any external funding, and informed consent was waived due to its retrospective nature. As the largest tertiary care center in Qatar, WWRC recorded 70,000 births during the study period, with 700 ELBW infants admitted to the neonatal intensive care unit (NICU). Dexamethasone was administered in our practice to infants with evolving or established BPD to facilitate extubation, based on the discretion of the neonatologist regarding the timing of initiation and dosage. The utilization of other systemic PNS such as hydrocortisone was rarely employed in our unit during the study period except as a vasopressor for hypotension.

### Data collection

The main objective of this study was to assess the achievement of successful extubation in ELBW with either evolving or established BPD after administering dexamethasone treatment. In this study, we employed Jensen's definition of BPD (1). Data on successful extubation were collected within 72 h and seven days after treatment initiation, by course completion, and within seven days after treatment completion. Secondary objectives were to evaluate for significant neonatal outcomes in the NICU, which included respiratory outcomes, death, and other significant neonatal morbidities. Neonatal data included gestational age (GA), gender, BW, small for gestational age (SGA), ethnicity, APGAR scores, and discharge weight. Respiratory characteristics of the study infants included surfactant administration, mechanical ventilation for 28 days, mode of ventilation at 36 weeks post-menstrual age (PMA), total invasive and noninvasive ventilation days, and mean fraction of inspired oxygen (FiO_2_). Physicians collected obstetric data by reviewing mothers’ charts for clinical and demographic information, as well as pharmacological information from the pharmacy database. Maternal data included demographics, ethnicity, antenatal steroids administered, mode of delivery, preterm premature rupture of membranes (PPROM), oligohydramnios, chorioamnionitis, GBS status, and illness (maternal hypertension and gestational diabetes). Information pertaining to the course encompassed details such as the examethasone dose administered per kilogram, the total number of courses undertaken, the day of life at which the course was initiated, the duration of the course, and the cumulative dose administered. This comprehensive dataset was complemented by an analysis of respiratory parameters both before and after the initiation of each course. Ventilator-associated pneumonia (VAP), sepsis, necrotizing enterocolitis (NEC), and intestinal perforation that resulted in interruption or treatment of the medication were recorded. A secondary medical outcomes included VAP, sepsis, NEC, retinopathy of prematurity (ROP), intraventricular hemorrhage (IVH), periventricular leukomalacia (PVL), neurodevelopment impairment (NDI), grade of BPD, death, and death or BPD. In our study, neurodevelopmental assessments were conducted on infants aged between 18 and 24 months’ corrected age using both the Bayley Infant Neurodevelopmental Screener (BINS) and additional neurologic examinations, including the Alberta Infant Motor Scale (AIMS). NDI in our study was specifically defined as any level of risk identified by the BINS and AIMS across its four domains, which encompass neurologic integrity including motor, cognitive, and language deficits.

### Statistical analysis

Descriptive analyses were conducted to evaluate patient characteristics and clinical variables. Chi-square or Fisher's exact test were employed for binary variables to analyze differences between groups (low-dose and enhanced low-dose dexamethasone). Non-parametric continuous variables were analyzed using the Mann–Whitney *U*-test. Mean and standard deviation (SD) or median and interquartile range (IQR) were used to present continuous variables, while numbers and percentages were used for categorical variables. Stepwise logistic regression (backward) was conducted to examine the associations between various exposures and successful extubation at different time points after controlling for potential confounders. The multivariable model included all significant variables associated with successful extubation identified in the univariate analyses with a significance of 0.1. All *p*-values were two-tailed, and values below 0.05 were considered statistically significant. Statistical analyses were performed using SPSS for Windows (version 29.0, IBM Corp., Armonk, NY, USA).

## Results

We evaluated 94 children who received dexamethasone treatment in the NICU for inclusion in the study. After excluding 17 infants, the study included 77 patients who received a total of 121 doses of dexamethasone (Figure 1). The characteristics of study subjects are summarized in Table 1. The study population was divided between those who received low-dose dexamethasone (*n* = 49) vs. enhanced low- dose dexamethasone (*n* = 28). The GA of neonates in the low-dose group was 24.9 ± 1.6 weeks vs. 25.3 ± 1.2 weeks in the enhanced low-dose group. The BW of neonates in both groups was respectively 748 ± 114 vs. 765 ± 124 g. Majority of patients were male in both groups (65% vs. 71%; *p* = 0.581). There were various ethnicities amongst the groups such as African (6.1% vs. 10.7%) and European (8.3% vs. 4.6%) without any visible differences. However, Asian population was much higher in the enhanced low-dose group 42.9% vs. low-dose 22.4%, while Middle Eastern ethnicity was higher in the low-dose group 63.3% vs. 42.9%. Difference in APGAR scores at 1 and 5 min showed no statistical significance between the groups. Discharge weight was significantly higher in the enhanced low-dose dexamethasone group compared to low-dose group (4,080 vs. 3,305 g; *p* = 0.048).

**Table 1:** Neonatal and maternal characteristics of infants received dexamethasone regimens.

	Low dose (*n* = 49)	Enhanced low dose (*n* = 28)	*P*
Neonatal			
GA–weeks (mean, SD)	24.9 ± 1.6	25.3 ± 1.2	0.064
BW–grams (mean, SD)	748 ± 114	765 ± 124	0.433
SGA	3 (6.1%)	2 (7.1%)	0.999
Male gender	32 (65%)	20 (71%)	0.581
Race			0.181
African	3 (6.1%)	3 (10.7%)	
Asian	11 (22.4%)	12 (42.9%)	
European	4 (8.3%)	1 (4.6%)	
Middle Eastern	31 (63.3%)	12 (42.9%)	
APGAR score (1 min), median (IQR)	5 (2–6)	5 (3–6)	0.970
APGAR score (5 min), median (IQR)	8 (7–8)	8 (7–9)	0.303
Discharge DOL, median (IQR)	141 (97–178)	155 (107–202)	0.194
Discharge PMA, median (IQR)	53.9 (39–50.6)	48.2 (41.8–55.1)	0.120
Discharge weight-grams (mean, SD)	3,305 ± 1,510	4,080 ± 1,757	0.048
SGA at discharge	30 (81%)	16 (61.5%)	0.085
Maternal			
Maternal age- years (mean, SD)	29.1 ± 6.6	31.2 ± 7.2	0.334
Vaginal delivery	23 (46.9%)	15 (53.6%)	0.575
Maternal hypertension	6 (12.2%)	6 (21.4%)	0.336
Gestational diabetes	11 (22.5%)	6 (21.4%)	0.999
GBS status, positive	5 (10.6%)	6 (21.4%)	0.311
Chorioamnionitis	9 (18.4%)	5 (17.8%)	0.955
Oligohydramnios	7 (14.3%)	6 (21.4%)	0.430
PROM	16 (32.6%)	8 (28.6%)	0.710
Multiple gestations	17 (34.7%)	4 (14.3%)	0.053
Antenatal steroid (*n* of doses)			0.727
0	4 (8.2%)	1 (3.6%)	
1	14 (28.6%)	8 (28.6%)	
≥2	31 (63.3%)	19 (67.9%)	
Multiparity	13 (26.5%)	9 (32%)	0.600

DART, dexamethasone: a randomized trial; GA, gestational age; BW, birth weight; SGA, small for gestational age; DOL, day of life; PMA, post-menstrual age; PROM, premature rupture of membranes; *n*, number of participants. Continuous variables expressed as means and standard deviations (SD) or median and interquartile range (IQR) as appropriate.

Maternal characteristics between the groups were further analyzed in Table 1 with a median age of mothers being 29.1 ± 6.6 in the low-dose group vs. 31.2 ± 7.2 enhanced low-dose group. Multiple gestations were much higher in the low-dose group 34.7% vs. 14.3% in the enhanced low-dose group, while not significant *p* = 0.053. Medical conditions during pregnancy were also analyzed including maternal hypertension (12.2% vs. 21.4% *p* = 0.311), gestational diabetes (22.5% vs. 21.4%; *p* = 0.999) and positive GBS screening (10.6% vs. 21.4%; *p* = 0.2), with no statistical significance between the groups. Other maternal factors examined were chorioamnionitis, PPROM, and oligohydramnios, did not show significant difference. The majority of mothers in both groups received at least one dose of antenatal steroids, with similar percentages in both groups, 91.7% vs. 96.4%. Additionally, nearly two-thirds of mothers received 2 doses of antenatal steroids in each group.

The respiratory characteristics of infants that received dexamethasone regimens were analyzed between the groups in Table 2. No statistical significance was found between the number of surfactant doses received whether none, one or ≥ two used between the two groups (*p*-value = 0.156). Receiving mechanical ventilation was assessed at 28 days between the low-dose and enhanced low-dose (83.7% vs. 92.8%; *p* = 0.312) and at 36 weeks PMA (22.5 vs. 22.2; *p* = 0.859), showed no statistical significance. No statistical significance was also observed between median invasive ventilation days (*p* = 0.787). However, the low-dose group received more non-invasive ventilation days compared to the enhanced low-dose group (40 vs. 60; *p*-value = 0.026). Additionally, infants who received dexamethasone therapy in the first 28 days were 37.4% vs. 25%; *p* = 0.377. Other respiratory characteristics analyzed were median FiO_2_ at 28 days and at 36 weeks (40 vs. 57; *p*-value = 0.210) and (25 vs. 29; *p*-value = 0.08), respectively. The proportions of infants who received repeated courses of dexamethasone (38.8% vs. 39.3%; *p*-value = 0.965), showed no differences between the two groups.

**Table 2:** Respiratory characteristics of infants that received dexamethasone regimens.

	Low dose (DART) (*n* = 49)	Enhanced low dose (*n* = 28)	*P*
Surfactant doses			0.156
0	0 (0.0%)	2 (7.1%)	
1	19 (38.8%)	9 (32.1%)	
≥2	30 (61.2%)	17 (60.7%)	
Mechanical ventilation for 28 days	41 (83.7%)	26 (92.8%)	0.312
Mechanical ventilation at 36 weeks PMA	9 (22.5%)	6 (22.2%)	0.859
Invasive ventilation days, median (IQR)	46 (33.5–65.5)	47 (33.3–70.3)	0.787
Noninvasive ventilation days, median (IQR)	40 (13–71)	60 (35.8-90.5)	0.026
Dexamethasone before 28 days	17 (34.7%)	7 (25%)	0.377
FiO_2_ at 28 days (%), median (IQR)	40 (30–60)	57 (36–60)	0.210
FiO_2_ at 36 weeks (%), median (IQR)	25 (23–30)	29 (24–50)	0.080
Multiple courses of dexamethasone	19 (38.8%)	11 (39.3%)	0.965

DART, dexamethasone: a randomized trial; PMA, post-menstrual age; FiO_2_, fraction of inspired oxygen. Continuous variables expressed as means and standard deviations (SD) or median and interquartile range (IQR) as appropriate.

Results of respiratory outcomes associated with different dexamethasone regimens are summarized in Table 3. The low-dose group were administered 75 courses vs. 46 in the enhanced low-dose group. The dosing weight for all courses was 1,696 ± 1,174 g in the low-dose group vs. 1,907 ± 1,280 g in the enhanced low-dose group *p* = 0.244. The dosing weight in grams at the start of the first dose was also evaluated and showed no significant differences between the groups *p* = 0.154. Day of life in terms of all courses and first course were analyzed and showed no significance, *p* = 0.571 and *p* = 0.603, respectively. Assessment of PMA in weeks at the start of all doses showed no significant difference between the groups (33.7 ± 6.7 vs. 35.3 ± 6.6; *p* = 0.112). However, PMA at the first course was found to be significant amongst the groups (30.8 ± 3.4 vs. 32.1 ± 2.5; *p* = 0.014). The median course dose per kg (mg) received was found to be higher in the enhanced low-dose group (0.73 vs. 1.27; *p* = <0.001). Also, evaluation of the median course duration (in days) between the groups was lower in the low-dose group (7 vs. 10. days *p* = <0.001). Statistical significance was observed in the course cumulative doses (mg) between the groups, with the enhanced low-dose group having a higher median dose (0.79 vs. 1.65; *p* = 0.001), and a higher total cumulative doses (mg) received throughout the NICU stay (1.00 vs. 1.99 (*p* < 0.001).

**Table 3:** Respiratory outcomes associated with dexamethasone regimens.

	Low dose (DART) (*n* = 75)	Enhanced low dose (*n* = 46)	*P*
Dosing weight (all courses) (g), mean ± SD	1,696 ± 1,174	1,907 ± 1,280	0.244
Dosing weight (first course) (g), mean ± SD	1,185 ± 487	1,293 ± 416	0.154
Day of life at start (all courses), mean ± SD	56.4 ± 43	60.5 ± 45	0.571
Day of life at start (first course), mean ± SD	36.3 ± 20.4	37.4 ± 15	0.603
PMA at start (all course) (wks), mean ± SD	33.7 ± 6.7	35.3 ± 6.6	0.112
PMA at start (first course) (wks), mean ± SD	30.8 ± 3.4	32.1 ± 2.5	0.014
Course duration (days), median (IQR)	7 (3–10)	10 (8–14)	<0.001
Dose (mg/kg), median (IQR)	0.73 (0.53–0.86)	1.27 (0.97–2.05)	<0.001
Course cumulative dose (mg), median (IQR)	0.79 (0.57–1.00)	1.65 (1.12–2.17)	<0.001
Total cumulative dose (mg), median (IQR)^a^	1.00 (0.70–1.55)	1.99 (1.22–4.03)	<0.001
FiO_2_ before course (%), median (IQR)	45 (35–80)	63 (45–90)	0.042
FiO_2_ after course (%), median (IQR)	35 (0.73–0.86)	40 (30–65)	0.527
On invasive ventilation	68 (91%)	45 (97.8%)	0.124
Extubated within 72 h of initiation^b^	17 (25%)	10 (22.2%)	0.735
Extubated within 7 days of initiation^b^	28 (41.2%)	14 (31.1%)	0.278
Extubated by the end of treatment course^b^	31 (45.6%)	24 (53.3%)	0.420
Extubated by 7 days post treatment^b^	37 (54.4%)	29 (64.4%)	0.289
Extubation day of life, mean ± SD	49.3 ± 30.6	63.2 ± 44.2	0.174
Any respiratory weaning after course^c^	42 (56%)	30 (65.2%)	0.316
VAP during treatment	16 (21.3%)	14 (30.4%)	0.260
NEC during treatment	1 (1.3%)	0 (0.0%)	0.999
Sepsis during treatment	10 (13.3%)	4 (8.7%)	0.439

DART, dexamethasone: a randomized trial; G, grams; Wks, weeks; PMA, post-menstrual age; Kg, kilogram; Mg, milligrams; FiO_2_, fraction of inspired oxygen. VAP, ventilator associated pneumonia; NEC, necrotizing enterocolitis. Continuous variables expressed as means and standard deviations (SD) or median and interquartile range (IQR) as appropriate.

^a^ Total dexamethasone dose received during hospitalization.

^b^ Cumulative numbers of extubated infants who received dexamethasone treatment, excluding non-invasive support cases (*n* = 8).

^c^ Respiratory weaning after completing a course refers to any alteration in ventilation mean airway pressure by ≥20% or a reduction in FiO_2_ based on the following conditions: a decrease of ≥25% if the initial FiO_2_ was <60%, or a decrease of ≥40% if the initial FiO_2_ was ≥60%. This also applies whenever the FiO_2_ is decreased to <25%.

Other parameters assessed in Table 3 included the median baseline FiO_2_ (%) before dexamethasone course and it was significant between the groups (45 vs. 63; *p* = 0.042). On the other hand, median FiO_2_ (%) after dexamethasone course showed no significance (35 vs. 40; *p* = 0.527). The vast majority (>90%) of patients in each group were on invasive ventilation, and there was no difference between the groups (*p* = 0.124). The proportions of infants extubated at different time points from dexamethasone treatment initiation were assessed between the low-dose and enhanced low-dose groups. There were no significant differences between the groups in extubation rates within 72 h (25% vs. 22.2%; *p* = 0.735) or following seven days from initiating therapy (41.2% vs. 31.1%; *p* = 0.278). Further analysis of potential extubation either at the end of treatment course or seven days post treatment showed no statistical difference between the groups (45.6% vs. 53.3%; *p* = 0.420 and (54.4% vs. 64.4%; *p* = 0.389). Reduction in respiratory support of any kind was not significant between the groups, *p* = 0.316. Infants who received enhanced low-dose were extubated at a mean age of 63 vs. 49 days of life compared to infants who received a lower dose of dexamethasone; however, this finding was not statistically significant (*p* = 0.174). Both the low-dose and enhanced low-dose groups had a high but non-significant incidence of VAP during treatment (21.3% vs. 30.4%; *p* = 0.260) and sepsis (13.3% vs. 8.7%; *p* = 0.439), while the presence of NEC was low (1.3% vs. 0.0%; *p* = 0.999), respectively.

Secondary outcomes associated with dexamethasone regimen usage were also analyzed throughout the study and are summarized in Table 4. Several neonatal-specific factors were assessed between the low-dose and enhanced low-dose groups, including VAP (61% vs. 61%; *p* = 0.965), sepsis (45% vs. 50%; *p* = 0.666), NEC (18.4% vs. 7.1%; *p* = 0.310), any ROP (71.4% vs. 74%; *p* = 0.805), severe ROP (40.8% vs. 37%; *p* = 0.747), treated ROP (8.8% vs. 19%; *p* = 0.408), medically treated PDA (53% vs. 53.6%; *p* = 0.966), with only two infants in each group requiring device closure of the PDA, and moderate and severe BPD (85.4% vs. 100%; *p* = 0.074). Additional analysis of long-term outcomes such as NDI showed no significant difference between the groups (30.7% vs. 29.1%; *p* = 0.893). There were 13 (16.9%) death cases during the NICU hospitalizations (22.4% vs. 7.1%; *p* = 0.117), with a median day of death of 92 vs. 145 days, *p* = 0.194 in the low-dose and enhanced low-dose groups. Infants who died before and after 36-weeks PMA were four and seven in the low-dose group and one and one in the enhanced low-dose group. Additionally, the incidence of death or severe BPD was 30.6% vs. 25%; *p* = 0.6 between the two groups.

**Table 4:** Secondary outcomes associated with dexamethasone regimen usage.

	Low dose (DART) (*n* = 49)	Enhanced low dose (*n* = 28)	*P*
VAP	30 (61%)	17 (61%)	0.965
Sepsis	22 (45%)	14 (50%)	0.666
NEC	9 (18.4%)	2 (7.1%)	0.310
ROP, any	35 (71.4%)	20 (74%)	0.805
ROP, severe	20 (40.8%)	10 (37%)	0.747
ROP, treated	3 (8.8%)	4 (19%)	0.408
PDA, medical	26 (53%)	15 (53.6%)	0.966
PDA, closure	2 (4%)	2 (7.1%)	0.619
BPD, moderate-severe^a^	35 (85.4%)	27 (100%)	0.074
IVH, any	17 (36.2%)	8 (28.6%)	0.500
IVH, severe	9 (19.1%)	4 (14.2%)	0.756
PVL, any	2 (4.3%)	1 (3.6%)	0.999
NDI^b^	12 (30.7%)	7 (29.1%)	0.893
Death^c^	11 (22.4%)	2 (7.1%)	0.117
Death, day of life, median (IQR)	92 (54–171)	145 (71–145)	0.194
Death or severe BPD	15 (30.6%)	7 (25%)	0.600

DART, dexamethasone: a randomized trial; VAP, ventilator associated pneumonia; NEC, necrotizing enterocolitis, ROP, retinopathy of prematurity; PDA, patent ductus arteriosus; IVH, intraventricular hemorrhage; PVL, periventricular leukomalacia; NDI, neurodevelopmental impairment; BPD, bronchopulmonary dysplasia; PNS, prenatal steroids. Continuous variables expressed as means and standard deviations (SD) or median and interquartile range (IQR) as appropriate.

^a^ Among the 72 surviving infants at 36 weeks post-menstrual age.

^b^ Excluding death (*N* = 13) and lost to follow-up (*N* = 1).

^c^ Total deaths before 36 weeks post-menstrual age (*N* = 5, DART 4/11 and enhanced low dose 1/2 cases).

Univariate and stepwise multivariate regression analyses were conducted to identify significant variables associated with successful extubation at various time points: 72 h post dexamethasone treatment, within seven days of treatment initiation, by the end of dexamethasone treatment, and within seven days of dexamethasone treatment completion, as summarized in Table 5.

**Table 5:** Regression analysis of variables associated with successful extubation at various time points following initiation of dexamethasone treatment.

Univariate					Multivariate			
	OR	95% C.I.		*P*	aOR	95% C.I.		*P*
		Lower	Upper			Lower	Upper	
Logistic regression analysis of significant factors associated with extubation within 72 h of dexamethasone treatment initiation^a^								
Baseline FiO_2_	0.935	0.904	0.967	<.001	0.951	0.920	0.984	0.004
Invasive ventilation duration	0.979	0.968	0.989	<.001				
Course duration	0.742	0.642	0.857	<.001	0.840	0.721	0.980	0.026
Logistic regression analysis of significant factors associated with extubation within 7 days of dexamethasone treatment initiation^a^								
Baseline FiO_2_	0.949	0.928	0.970	<.001	0.966	0.941	0.992	0.011
Invasive ventilation duration	0.980	0.970	0.991	<.001	0.962	0.939	0.987	0.003
Ethnicity, Middle Eastern	3.017	1.382	6.828	0.006	0.573	0.299	1.097	0.093
Dose per Kg	2.080	1.070	4.044	0.031				
Cumulative dose	0.946	0.889	1.007	0.082	1.864	1.270	2.736	0.001
Course duration	0.759	0.673	0.857	<0.001	0.759	0.652	0.884	<0.001
Logistic regression analysis of significant factors associated with extubation by the end of dexamethasone treatment^a^								
Baseline FiO_2_	0.964	0.948	0.981	<.001	0.971	0.948	0.994	0.016
Invasive ventilation duration	0.980	0.970	0.991	<.001	0.977	0.961	0.993	0.005
Parity	1.227	0.948	1.587	0.121				
Ethnicity, Middle Eastern	2.283	1.074	4.849	0.032				
Dose per Kg	2.067	1.078	3.096	0.028	11.71	3.070	44.69	<0.001
Cumulative dose	0.953	0.905	1.004	0.070				
Course duration	0.931	0.858	1.010	0.084	0.852	0.736	0.987	0.033
Logistic regression analysis of significant factors associated with extubation within 7 days of dexamethasone treatment completion^a^								
Baseline FiO_2_	0.960	0.943	0.976	<.001	0.960	0.938	0.982	<0.001
Invasive ventilation duration	0.976	0.966	0.987	<.001	0.974	0.959	0.989	<0.001
Parity	1.593	1.140	2.225	0.006				
Ethnicity, Middle Eastern	2.549	1.179	5.512	0.017				
Maternal age	1.077	1.009	1.149	0.026				
Dose per Kg	2.040	1.011	4.115	0.047	7.36	2.421	22.37	<0.001
Gravidity	1.148	0.976	1.351	0.095				
Cumulative dose	0.93	0.880	0.983	0.010				

aOR, adjusted odds ratio; CI, confidence interval; FiO_2_, fraction of inspired oxygen. Significant *P* < 0.05.

^a^ Factors not significantly associated with outcome in univariate analysis (did not enter multivariate regression analysis): birth weight, gestational age, gender, small for gestational age, birth order, multiple births, premature rupture of membranes, mode of delivery, Apgar scores, chorioamnionitis, maternal group B streptococcus status, gestational diabetes, hypertensive disorder during pregnancy, administration of antenatal steroids, gender, order of dexamethasone initiation course, timing of dexamethasone administration, post-menstrual age at dexamethasone initiation, and total duration of non-invasive respiratory support.

Among the demographic and clinical variables examined, the following predictors were found to be associated with successful extubation at 72 h post dexamethasone treatment initiation in the univariate regression model: baseline FiO_2_, duration of invasive ventilation, and course duration. However, in the multivariate analysis, only baseline FiO_2_ (adjusted odds ratio [aOR]: 0.951, 95% confidence interval [CI]: 0.920–0.984, *p* = 0.004) and course duration (aOR: 0.840, CI: 0.721–0.980, *p* = 0.026) remained significantly associated with successful extubation at this time point.

Similarly, for successful extubation at seven days post treatment initiation, the univariate regression model identified the following predictors: baseline FiO_2_, duration of invasive ventilation, ethnicity, dose per kg, cumulative dose, and course duration. In the multivariate analysis, baseline FiO_2_ (aOR: 0.966, CI: 0.941–0.992, *p* = 0.011), duration of invasive ventilation (aOR: 0.962, CI: 0.939–0.987, *p* = 0.003), cumulative dose (aOR: 1.864, CI: 1.270–2.736, *p* = 0.001), and course duration (aOR: 0.759, CI: 0.652–0.884, *p* < 0.001) remained significant.

Additionally, regarding extubation by the end of the dexamethasone course treatment, out of the significant predictors found in the univariate regression analysis (baseline FiO_2_, duration of invasive ventilation, parity, ethnicity, dose per kg, cumulative dose, and course duration), only baseline FiO_2_ (aOR:0.971, CI: 0.948–0.994, *p* = 0.016), dose per kg (aOR:11.71, CI: 3.070–44.691, *p* < 0.001), duration of invasive ventilation (aOR:0.977, CI: 0.961–0.993, *p* = 0.005), and course duration (aOR:0.852, CI: 0.736–0.987, *p* = 0.033) remained significant in the multivariate analysis.

Finally, the regression analysis within seven days of completing dexamethasone treatment showed a significant association in the univariate analysis for several factors, including baseline FiO_2_, duration of invasive ventilation, parity, ethnicity, maternal age, dose per kg, gravidity, and cumulative dose. From these predictors, baseline FiO_2_ (aOR:0.960, CI: 0.93–0.982, *p* < 0.0001), duration of invasive ventilation (aOR:0.974, CI: 0.95–0.989, *p* < 0.001), and dose per kg (aOR:7.361, CI: 2.42–22.37, *p* < 0.001) remained significant in the multivariate analysis.

## Discussion

BPD remains a challenging respiratory outcome among ELBW neonates in the NICU (1–4). The administration of PNS can be considered as a crucial therapy for these neonates to improve parenchymal lung disease and facilitate extubation (3–6, 8–10). Although DART protocol is the mainstay therapy in our unit, higher dose regimens such as enhanced low-dose dexamethasone, are currently used on a case-by-case basis by neonatologists. This practice aligns with the common approach of utilizing this dose of dexamethasone (DART protocol) for evolving or established BPD suggested by Doyle LW et al. (23). The same author conducted a meta-analysis of 23 RCTs on the use of systemic PNS for the prevention of BPD in preterm neonates (27). Their analysis revealed that two-thirds of the studies included in the meta-analysis utilized dexamethasone (*n* = 21) for BPD prevention.

Neonates in both groups showed similar neonatal-specific characteristics. However, it was observed that neonates receiving the enhanced low-dose dexamethasone regimen had a higher weight at the time of hospital discharge, required higher FiO_2_, and remained longer on non-invasive respiratory support. Furthermore, they were extubated later compared to infants who received a lower dose of dexamethasone, although this latter finding was not statistically significant. These observations suggest a more severe manifestation of BPD. Thus, providing a potential explanation for the increased utilization of the enhanced low-dose dexamethasone regimen by attending neonatologists in this group. According to our study, the enhanced low-dose dexamethasone regimen resulted in a significantly longer duration of therapy and a higher cumulative dose compared to the low-dose dexamethasone protocol. This was expected as the enhanced low-dose regimen involves a higher overall dose and/or a longer duration of therapy. Bonnie L Marr et al. conducted a randomized controlled trial (RCT) in which they compared a 42-day course of dexamethasone to a nine-day course for the treatment of BPD in extremely preterm neonates (28). The study found that the 42-day group had a significantly higher overall dose of dexamethasone compared to the 9-day group (*P* < 0.001). These findings are expected with all studies that utilize a higher dexamethasone dose than the standard DART protocol dose.

Our study findings showed no difference between the groups in terms of development of any adverse outcomes including NDI at 18–24 months. These findings suggest that the safety profile of dexamethasone at an enhanced low-dose is comparable to that of the low-dose (DART protocol). A meta-analysis by Ramaswamy et al. assessed the safety of 14 different PNS regimens used for the prevention of BPD in neonates and secondary outcomes included NDI (18–24 months) (29). It was found that moderately early (2–4 mg/kg) of dexamethasone was potentially the most appropriate regimen for preventing BPD. Also, none of the interventions were associated with an increased or decreased risk of NDI. Linan Zeng et al., conducted a meta-analysis which included 47 RCTs with 6,747 participants and focused on the efficacy and safety of high dose dexamethasone (>3 mg/kg) compared to other corticosteroids for prevention of BPD in preterm infants (30). The meta-analyses concluded that higher-doses are effective, but not without the potential increased risk of adverse neurodevelopment effects. A systematic review conducted in 2017 by Onland W. et al., which compared different corticosteroid regimens on mortality and neurodevelopmental outcomes (31). In a subgroup analysis of the study, it was concluded that there were no differences between high and low-dose regimens on death or any abnormal neurodevelopmental outcomes. Other studies have suggested that the use of dexamethasone at various high doses could potentially be harmful for overall brain development (32, 33). In our study, enhanced low-dose dexamethasone, which is considered a lower cumulative dose compared to those referred to as “moderate to high” in the previously mentioned studies carries a considerably less risk for NDI at 18–24 months as suggested by the results. However, a longer-term follow-up beyond two years on NDI is warranted to further confirm the findings of our study.

Our study did not show that an enhanced low-dose dexamethasone is associated with a higher rate of extubation at various time points. Nonetheless, regression analysis showed that a higher dose per kilogram or higher cumulative dose with a shorter duration are associated with successful extubation. These findings are supported by recommendations by Zeng et al, who suggested aggressive initiation seems more effective (30). However, higher doses for longer durations are not recommended based on safety. Moreover, the regression analysis also showed that enhanced low dose dexamethasone dose significantly correlated with higher chances of neonatal extubation within different time frames of receiving dexamethasone course. This is an essential finding as it further sheds light on the potential importance of increasing dexamethasone dosing within ranges that are still considered to be safe (34). Studies have found similar findings where moderately high doses of dexamethasone were associated with a significant improvement in FiO_2_ (30, 35, 36). Despite the higher extubation rates associated with the enhanced low dose in our study, no differences were found between the rates of BPD outcomes between the groups.

Similarly, death was not clinically significant although higher in the low dose dexamethasone group. These findings are supported by a meta-analysis and other studies which showed no difference of composite death between various dexamethasone regimens (26, 37, 38).

This study has several strengths as it is the first of its kind to be conducted in Qatar. It also provides valuable insight on the use of dexamethasone in ELBW neonates with BPD as it compares two different dose regimens low dose (DART) and enhanced low-dose. The study focused on a vulnerable and specific population, ELBW neonates, for whom the optimal recommended dose of dexamethasone is still in question. In addition, matching was conducted to help control for potential confounding factors by matching patients in terms of birth weight and gestational age. This enhances the validity of the comparison between the groups. Regression analysis was used to help identify any predictors of successful extubation. This statistical approach adds depth to the findings by highlighting factors that influence extubation outcomes in ELBW infants. The consideration of secondary outcomes, such as death or BPD, provides a comprehensive view of the safety and efficacy of the treatments. Despite its strengths this study contained limitations such as its retrospective nature which has inherent biases with data collection and dependence on existing medical records. Recall bias also may potentially impact the accuracy of the data recorded. Although the study attempted to control for potential confounding factors however, some variables which were not accounted for could potentially influence the extubation outcomes. In addition, the study may be underpowered, due to the small sample size, to fully form conclusions on safety and efficacy outcomes. The need for further research to evaluate the findings of this study with larger sample sizes and longer follow-up data are warranted to help draw more clear conclusions.

## Conclusion

Our findings showed no significant differences in extubation success rates between the two regimens at various time points following treatment initiation. However, we identified important predictors of successful extubation, including baseline FiO_2_, course duration, and duration of invasive mechanical ventilation, which negatively impacted extubation success. Conversely, received dose per kilogram and cumulative dose positively correlated with successful extubation at different time points. These results underscore the need for a personalized approach to dexamethasone therapy in ELBW infants with BPD, considering individual patient characteristics and clinical factors.

## Data Availability

The raw data supporting the conclusions of this article will be made available by the authors, without undue reservation.
